# The Role of Hemoclips Reinforcement in the Ligation-Assisted Endoscopic Enucleation for Small GISTs in Gastric Fundus

**DOI:** 10.1155/2014/247602

**Published:** 2014-05-07

**Authors:** Ge Nan, Sun Siyu, Wang Sheng, Liu Xiang, Guo Jintao

**Affiliations:** The Shengjing Hospital, China Medical University, No. 36 Sanhao Street, Shenyang, Liaoning 110004, China

## Abstract

*Background.* Endoscopic ultrasonography- (EUS-) assisted band ligation has been proven to be a safe and effective procedure for the treatment of small gastrointestinal stromal tumors (GISTs) apart from the relatively high risk of the postligation perforation of the gastric fundus. The aim of this study is to investigate the efficacy of hemoclip reinforcement in treating small GISTs in the gastric fundus. *Method.* During a standard endoscopy, a transparent cap attached to the endoscopic tip was placed over the lesion to exert sustained maximal aspiration before a rubber band was released. Once a definite ligation was confirmed by EUS, the tumor was enucleated. Four to 6 hemoclips were placed on the folds around the ligation band to reduce the tension of the ligation site. *Results.* The small GISTs were resected completely in 192 patients. Two cases of delayed perforation were found 72 hours after the procedure and successfully treated with an ordinary conservative method. *Conclusion.* Hemoclip-reinforced endoscopic band ligation with systematic follow-up using EUS appears to be a simple and effective technique for the resection of small GISTs in the gastric fundus.

## 1. Introduction

GISTs are a type of neoplasm derived from gastrointestinal mesenchymal tissues and contain spindle cells and positive CD117 expression [1]. Currently, no therapeutic guideline is available for the treatment of asymptomatic small GISTs with a low degree of malignancy, as identified by EUS. The common approach to the management of small GISTs is periodical follow-up examination with EUS after endoscopic resection. Sun et al. [2–4] have reported on the application of EUS-assisted band ligation to treat small GISTs. Although this technique has been proven to be safe and effective, delayed perforation was recently reported as a severe complication, especially for GISTs localized in the gastric fundus [5]. The purpose of this study was to evaluate an improved ligation technique by placing 4 to 5 hemoclips around the ligation band to prevent perforation and complete the tumor enucleation above the band to obtain a pathological diagnosis. The study was approved by the Institutional Review Board of China Medical University.

## 2. Materials and Methods

### 2.1. Patients

**Figure 1:**
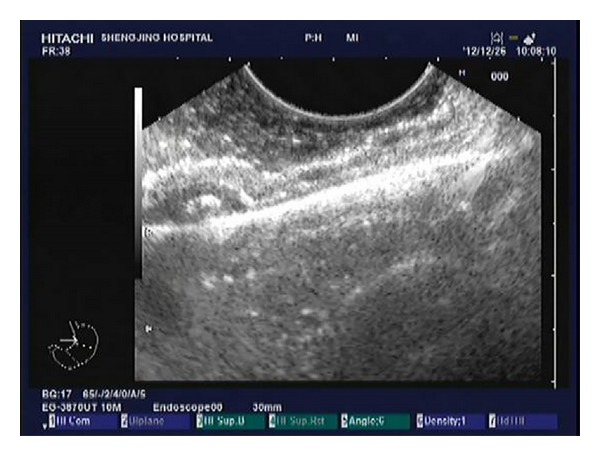
Lesion was localized to the muscularis propria of the gastric fundus, as confirmed by EUS.

A total of 192 patients with small tumors originating from the muscularis propria in the gastric fundus treated at Shengjing Hospital between May 2010 and June 2013 were included in this study. All patients gave informed consent for this procedure. Inclusion criteria were as follows. (1) Lesion was localized to the muscularis propria of the gastric fundus as confirmed by EUS (Figure 1). (2) Tumor diameter was no larger than 12 mm measured with EUS (corresponding to the diameter of the transparent hood used for ligation). (3) Patients did not take aspirin or other nonsteroid anti-inflammatory drugs for at least 1 week prior to the procedure. (4) Complete blood counts, prothrombin time, and partial thromboplastin time were normal for all patients.

### 2.2. Devices

Ligation was carried out with a conventional 2.7 mm channel endoscope (EG2770K, Pentax) and an air-driven band ligator (Sumitbe, Akita, Japan). EUS was performed with a curved linear-array echoendoscope (EG3830UT, Pentax Precision Instruments). Several hemoclips (standard size 8 mm open, Olympus Medical System) were placed around the band to reduce tension of the ligation site. The tumor was dissected by the triangle or hook knife (Olympus, Tokyo, Japan). The clips were further immobilized by spraying 1.5 mL medical adhesive (*α*-butyl cyanoacrylate spray, Beijing Suncon Medical Adhesive Co., Ltd.) on the surfaces.

### 2.3. Methods

**Figure 2:**
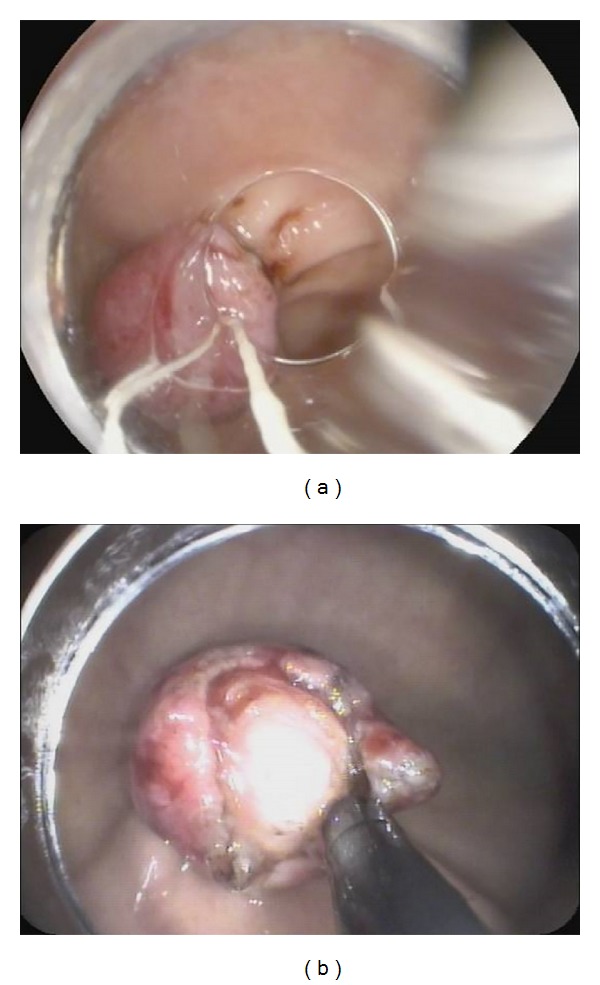
(a) The tumor was ligated completely. (b) A hook knife or triangle knife was used to cut the surrounding mucosa open above the band.

**Figure 3:**
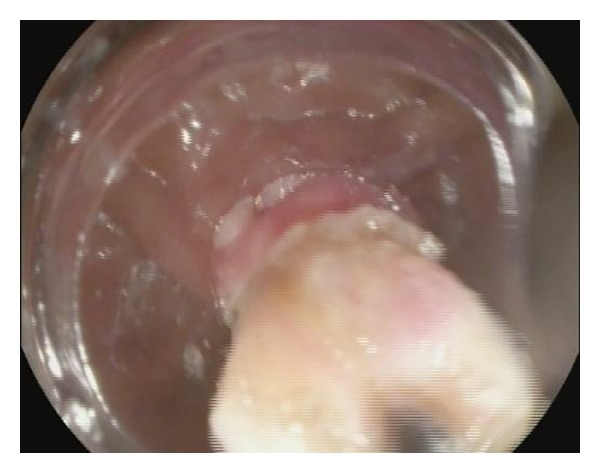
The tumor was dissected from the muscular layer and retrieved by the foreign body forceps.

**Figure 4:**
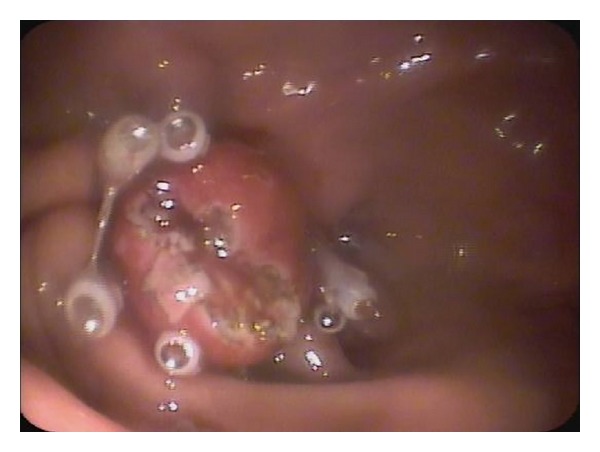
Four to 6 hemoclips were placed on the folds around the ligation band to reduce tension of the ligation site.

The ligation process was described by Sun et al. in detail [2]. The lesion was fully aspirated into a transparent cap attached to the endoscope that was introduced into the stomach under conscious sedation, using intravenous administration of propofol before the rubber band was released. EUS was used to determine the complete confinement of the hypoechoic mass within the rubber band. The band can cause an acoustic shadow. The hypoechoic structure (tumor) can be seen above band if aspiration was inadequate with only the mucosal and submucosal layers confined, and the band was removed using a foreign body forceps before the lesion was religated. After a definite ligation, a hook knife or triangle knife was used to cut the surface mucosa open until the tumor was identified (Figure 2). The tumor was dissected from the muscular layer and retrieved by the foreign body forceps for pathological and immunological examination (Figure 3). After the enucleation, 4 to 5 hemoclips were placed on the mucosal folds formed by the ligation (Figure 4) to reduce tension of the ligation site. The air in the stomach was fully sucked before the hemoclip was released. Then, medical adhesive was sprayed onto the surfaces of the clips and the wound surface, in order to immobilize the clips firmly. After the procedure, a gastrointestinal decompression tube was retained for 2 days. Patients received a proton pump inhibitor (PPI) once daily (Esomeprazole 40 mg) and endoscopic examination once weekly beginning 3 weeks after ligation until complete healing was achieved. Then, all patients discontinued PPI and underwent EUS every 2-3 months on schedule.

## 3. Results

The 192 patients (72 men and 120 women; mean age 56.3 years with a range of 30–73 years) with 192 small tumors localized to the muscularis propria of the gastric fundus were selected for hemoclip-reinforced ligation-assisted endoscopic enucleation. Only 32 patients experienced mild epigastric pain. Other digestive symptoms included heartburn (46 patients), abdominal distention (78 patients), and early satiety (19 patients). There was no evidence supporting causality between tumors and these symptoms. In all, 72 patients were asymptomatic and had their lesions discovered during screening examinations. All patients expected active intervention for their lesions. All the lesions (median diameter, 8 mm; range, 5–12 mm) were localized in the gastric fundus. All the lesions confirmed by EUS were completely ligated once, followed by tumor dissection. After the dissection, the continuity of the digestive tract was maintained by the ligation band. Hemoclips (mean number, 4 clips for each lesion; range, 3–6 clips) were placed around the ligation site.

All 192 tumors were resected completely. Histopathological evaluations were all confirmed with the diagnosis of GISTs (177/192) and leiomyoma (15/192). Immunohistochemical analysis showed c-kit/CD117 positive, CD34 positive, S100 negative, and smooth muscle actin negative results for GISTs. No immediate perforation or bleeding was found after the dissection.

**Figure 5:**
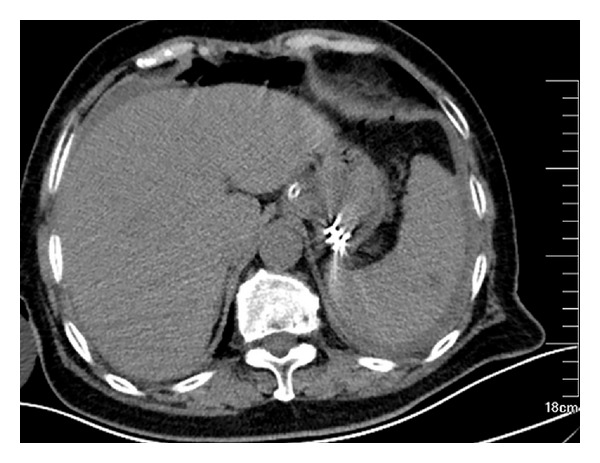
Emergent CT scanning showed a small perforation of the ligation site after the band exfoliation.

The most frequent clinical complaints after ligation included abdominal discomfort (114/192), abdominal distension (46/192), and nausea (31/192). The mean fasting period after ligation was 3 days and the median hospitalization time was 7 days. Obvious abdominal pain was found in two patients 72 hours after the procedure and directly following eating. Results for emergent CT scanning showed a small defect of the gastric wall at the ligation site after the band exfoliation (Figure 5). A little gas was found outside the ligation site without signs of peritoneal cavity infection. Both of the patients became febrile (38.5°C and 39°C) following the abdominal pain, with the WBC being raised above the normal level. Deep tenderness was elicited. In these patients the fasting time was extended by another 48 hours and the decompression tube in the stomach was maintained. Antibiotics and PPI were also given intravenously for 48 hours. Ordinary conservative treatment produced a favorable clinical outcome.

**Figure 6:**
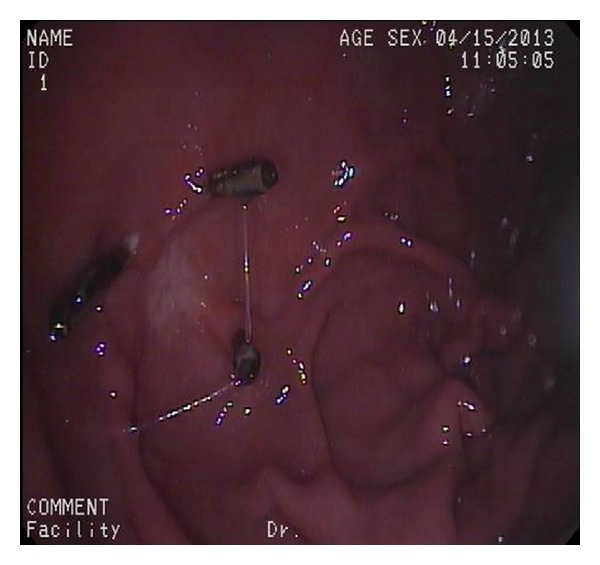
Complete healing of gastric mucosa after ligation.

The mean time to complete healing of the gastric mucosa after ligation was 4.5 weeks (range, 4–6 weeks) (Figure 6). The longest retention time of the hemoclips was 12 months, which was longer than expected, and which were removed by the foreign body forceps during endoscopic surveillance.

## 4. Discussion

GISTs are the most common mesenchymal neoplasms of the gastrointestinal tract. Small GISTs are frequently asymptomatic and discovered incidentally during endoscopy or CT scanning for other indications [6, 7]. Since GISTs are considered to have only “malignant potential,” it is impossible to predict their metastasis. Because even tumors with a low mitotic index or those small in size may become metastatic, all GISTs should be resected once they are identified [8–10].

Traditionally, surgical resection is the method of choice for nonmetastatic GISTs. DeMatteo showed that survival is not affected by the microscopic margin of resection. Routine extensive lymphadenectomy is not warranted because lymphatic involvement is extremely rare in GISTs [11, 12]. Therefore, minimal invasive intervention including several endoscopic, laparoscopic, and combined techniques is a more rational choice for GISTs and has resulted in lower mortality and earlier recovery [13]. Endoscopy is the least invasive technique and has more advantages for elderly patients and those with high surgical risks, especially in treating small tumors.

Various methods including ESD and NOTES for complete endoscopic resection of GISTs have been reported. ESD technique is difficult to master because it requires thorough knowledge and skills in performing endoscopic hemostasis, wound closure, and use of devices under endoscope, so as to avoid severe complications. Sometimes, for GISTs tightly adherent to the muscularis propria, lesions have not been able to be resected completely by ESD [14]. Few resections have been done in the gastric fundus when the tumors are localized within muscularis propria. The fundic wall is very thin so that resection is often accompanied by simultaneous perforation.

Sun et al. have reported that small esophageal leiomyomas [2] GISTs, and duodenal stromal tumors could be resected safely using endoscopic band ligation techniques. The band sloughs spontaneously. When aspiration and band ligation were performed using a cap-mounted endoscope, all layers of the gastrointestinal tract and GISTs were ligated. The resultant ischemia and necrosis would cause sloughing of the band and the ligated gastric wall. The healing of the gastric wall is by gradual formation of serosal adhesion outside the band, in response to local inflammation. No perforation or other complications were reported, except for the tumors located in the gastric fundus [5]. As reported by Sun et al. in 2010, two patients with a GIST located at the gastric fundus complained of severe abdominal pain, one at 24 hours and one at 35 hours after the procedure. Upright abdominal pain films confirmed bilateral subphrenic free air, and both patients underwent emergent laparotomy. At operation, circular perforations of 2–2.5 cm diameter were found with gastric mucosa that appeared inverted. Those two cases of severe complications have limited the application of ligation technique in the gastric fundus.

In this study, a novel technique for complete resection of GISTs using hemoclip-reinforced ligation was demonstrated. GISTs were safely resected above rubber bands with 4 to 5 hemoclips placed on the folds around the ligation band. Medical adhesive was sprayed onto the surface to immobilize these clips.

Since all the reported gastric perforations occurred after endoscopic ligation at the fundus, premature sloughing of lesions may account for the perforation, although the exact histopathologic mechanisms of “all-layer” ligation is unknown. Premature sloughing of ligated gastric wall may result from the gastric wall being comparatively thin at this location. The band can often exfoliate prematurely during fundic ligation. Premature exfoliation of the band can cause perforation due to still-incomplete serosal adhesion. The sooner the band has exfoliated, the greater the risk of postprocedural complications is, including bleeding and perforation. We placed 4 to 5 hemoclips on the folds around the ligation band to reduce tension of the gastric wall and hoped this would be sufficient to delay the exfoliation of lesions, since perforation could be avoided when adhesion occurred ahead of exfoliation. In our study, two cases of perforation occurred 72 hours after the procedure, which was far longer than the cases reported in 2010 [5]. Also, the incomplete healing of the serosa revealed in CT scanning was not that large (compared with the 2.5 cm diameter perforation in 2010) and was only a small incompletely-healed defect. In our study, although the risk of perforation could still not be avoided, the patients with perforation did not suffer from complications of severe peritoneal cavity inflammation and require surgical intervention. The patients recovered with conservative treatment with a decompression tube and extended fasting time. The hemoclips may play an important role in the delayed sloughing time. The exact mechanism should be further tested by the animal study.

Hemoclips, originally developed 3 decades ago in Japan by Jensen and Machicado [15], have been used for endoscopic hemostasis since then and their use has been expanded to the management of many types of perforations and fistulas. However, their application in reinforcing band ligation has not previously been described. The use of small hemoclips can help reduce tension of the gastric wall, a much easier task than repairing a defect in the wall. Hemoclips placed around the ligation band could prevent premature exfoliation of the tumors effectively and sustain a decompressive effect, even after exfoliation of tumors.

It is also the first time that medical adhesive was used in strengthening ligation. Compound medical adhesive was originally developed for hemostasis and wound sealing. The main purpose of using medical adhesive in this procedure was to immobilize the hemoclips firmly and prevent their premature detachment from the gastric wall effectively. In this study, the retention time for these hemoclips ranged from 1 week to 1 year, much longer than the 2 weeks reported by Jensen et al. [16].

In conclusion, hemoclip-reinforced endoscopic band ligation with systematic follow-up using EUS appears to be a simple and effective technique for resection of small GISTs in the fundus by decreasing the risk of perforation. Although it cannot completely prevent perforation during the band exfoliation process, it can minimize a peritoneal cavity inflammation reaction and avoid the following necessary surgical intervention. Patients with GISTs located in gastric fundus should prolong the fasting time after the ligation.
